# Cellular hypoxia promotes osteogenic differentiation of mesenchymal stem cells and bone defect healing via STAT3 signaling

**DOI:** 10.1186/s11658-019-0191-8

**Published:** 2019-12-03

**Authors:** Xin Yu, Qilong Wan, Xiaoling Ye, Yuet Cheng, Janak L. Pathak, Zubing Li

**Affiliations:** 1 The State Key Laboratory Breeding Base of Basic Science of Stomatology (Hubei-MOST) and Key Laboratory of Oral Biomedicine, Ministry of Education, School and Hospital of Stomatology, Wuhan University, 237 Luoyu Road, Wuhan, 430079 China; 2 Department of Oral and Maxillofacial Trauma and Plastic Surgery, School and Hospital of Stomatology, Wuhan University, 237 Luoyu Road, Wuhan, 430079 China; 3 Department of Stomatology, Union Hospital, Tongji Medical College, Huazhong University of Science and Technology, Wuhan, 430022 China; 4 Key Laboratory of Oral Medicine, Guangzhou Institute of Oral Disease, Affiliated Stomatology Hospital of Guangzhou Medical University, Guangzhou, 510140 China

**Keywords:** Cellular hypoxia, Mesenchymal stem cells, STAT3-signaling, Osteogenic differentiation, Bone defect healing

## Abstract

**Background:**

Hypoxia in the vicinity of bone defects triggers the osteogenic differentiation of precursor cells and promotes healing. The activation of STAT3 signaling in mesenchymal stem cells (MSCs) has similarly been reported to mediate bone regeneration. However, the interaction between hypoxia and STAT3 signaling in the osteogenic differentiation of precursor cells during bone defect healing is still unknown.

**Methods:**

In this study, we assessed the impact of different durations of CoCl_2_-induced cellular hypoxia on the osteogenic differentiation of MSCs. Role of STAT3 signaling on hypoxia induced osteogenic differentiation was analyzed both in vitro and in vivo. The interaction between cellular hypoxia and STAT3 signaling in vivo was investigated in a mouse femoral bone defect model.

**Results:**

The peak osteogenic differentiation and expression of vascular endothelial growth factor (VEGF) occurred after 3 days of hypoxia. Inhibiting STAT3 reversed this effect. Hypoxia enhanced the expression of hypoxia-inducible factor 1-alpha (HIF-1α) and STAT3 phosphorylation in MSCs. Histology and μ-CT results showed that CoCl_2_ treatment enhanced bone defect healing. Inhibiting STAT3 reduced this effect. Immunohistochemistry results showed that CoCl_2_ treatment enhanced Hif-1α, ALP and pSTAT3 expression in cells present in the bone defect area and that inhibiting STAT3 reduced this effect.

**Conclusions:**

The in vitro study revealed that the duration of hypoxia is crucial for osteogenic differentiation of precursor cells. The results from both the in vitro and in vivo studies show the role of STAT3 signaling in hypoxia-induced osteogenic differentiation of precursor cells and bone defect healing.

## Introduction

Bone defect healing is a complex process involving numerous cellular signaling pathways mediated by multiple factors, including hypoxia, inflammation and mechanical loading. Resolving the clinical issues of delayed bone defect healing and fracture non-union requires a deeper understanding of these underlying cellular and molecular mechanisms.

In the early stage of bone defect healing, the hypoxia-inducible factor (HIF) regulatory pathway activates and further stimulates the expression of hypoxia response genes such as β-catenin and vascular endothelial growth factor (VEGF) [1–3]. Hypoxia in the vicinity of the bone defect triggers the osteogenic differentiation of precursor cells and promotes bone regeneration [2, 4–7]. Inducing hypoxia in precursor cells has been reported to enhance bone defect healing [3, 8–10]. Moreover, hypoxia promotes osteogenesis–angiogenesis coupling via VEGF signaling during bone defect healing [2, 11, 12]. Recently, hypoxia-based bone tissue engineering approaches have been reported to facilitate bone defect healing [12, 13]. However, the optimal duration of cellular hypoxia to achieve the maximum anabolic effect on osteogenic differentiation of MSCs is still unknown.

Signal transducer and activator of transcription 3 (STAT3) is a ubiquitously present transcription factor that mediates cell survival, proliferation and differentiation [14, 15]. STAT3 signaling plays a vital role in bone homeostasis. Osteoblast- and osteoclast-specific knockout of STAT3 significantly reduces bone mineral density in mice [16, 17]. Cellular hypoxia upregulates STAT phosphorylation in MSCs [18]. Similarly, an increase in STAT3 phosphorylation has been reported during the osteogenic differentiation of human periosteal progenitors. Sun et al. recently reported on the beneficial role of STAT3 signaling in bone defect healing via suppression of regulatory T cell function [19]. Osteoblast- and osteocyte-specific inactivation of STAT3 also decreases mechanical load-driven bone formation [20]. Akermanite bioceramics, an osteoinductive bone graft, upregulates STAT3 signaling and promotes bone defect healing. Cellular hypoxia enhances the migration of MSCs via STAT3 signaling [18]. Moreover, the upregulation of JAK2, an upstream of STAT3 signaling, has been reported to induce osteogenic differentiation of progenitor cells and bone defect healing [21].

Multiple findings from the literature indicate a possible role of STAT3 signaling in hypoxia-induced bone defect healing. However, the interaction between hypoxia and STAT3 signaling during the osteogenic differentiation of precursor cells and bone defect healing still needs to be investigated.

In this study, we assessed the impact of different durations of cellular hypoxia on the osteogenic differentiation of mesenchymal stem cells (MSCs). Furthermore, we investigated the role of STAT3 signaling in hypoxia-mediated osteogenic differentiation of precursor cells and bone defect healing.

## Materials and methods

### Isolation and culture of mice MSCs

Six-week old C57BL/6 male mice were obtained from the Wuhan University Centre for Animal Experiments. The Medical Ethics Committee of the College and Hospital of Stomatology of Wuhan University approved all the animal experiments performed in this study. The mice MSCs were isolated and cultured as described previously [21, 22].

### Animal study

All the animals in this study were treated according to internationally recognized guidelines on animal welfare. C57BL/6 mice (*n* = 75, male, 8 weeks old with a weight between 20 and 25 g) were used in this study. Femoral defects were created in 60 of the mice, while 15 mice were used as the blank control (i.e., no femoral injury). The 60 mice with femoral defects were randomly divided into 4 equal groups (control, CoCl_2_, CoCl_2_ + inhibitor, and inhibitor group) for the treatment protocol.

### Cell viability assay

For cell viability analysis, MSCs (3 × 10^3^ cells/well) were seeded in a 96-well plates (NEST Biotechnology). The full culture was for 7 days, with the cells being treated with CoCl_2_ (50 μM, Sigma) for 1, 3, 5 or 7 days. CoCl_2_ treatment in cell culture is an established method to create cellular hypoxia. CCK-8 reagent (10 μl) was added to each well with incubation for 4 h at 37 °C. The media were transferred to a new 96-well plate, and the absorbance was measured using a Thermo Fisher Scientific Microplate Reader at 450 nm.

HIF1-α is the main hypoxia-induced protein that gives further cellular signaling. CoCl_2_ treatment mimics hypoxia in cells by occupying the von Hippel-Lindau (VHL) protein-binding domain of HIF-α, thus preventing its degradation [23]. In our previous study, we tested various concentrations of CoCl_2_ and found that 50 μM robustly enhanced HIF-1α protein expression in mouse MSCs [18]. Therefore, in this study, we choose 50 μM CoCl_2_ treatment in MSC culture to create a hypoxic environment.

### Gene expression analysis

For quantitative real-time PCR assay, MSCs (1 × 10^6^ cells/well) were seeded in 6-well plates and cultured in osteogenic medium consisting of 50 mg/ml ascorbic acid (Sigma), 10^− 8^ mol/l dexamethasone (Sigma) and 10 mM β-glycerophosphate (Sigma). The cells were treated with 50 μM CoCl_2_ for 1, 3, 5 and 7 days. The STAT3 inhibitor cryptotanshinone (10 μM, Sigma) or DMSO was added to the cells in the appropriate groups for the duration of the culture. The dose of cryptotanshinone was chosen based on our previous research [18].

Tissue RNA was isolated from mice femurs with or without bone defects from each group (*n* = 3 per group) on day 7. The samples from mice without bone defects were used as the blank control. The samples from mice that had bone defects but that did not receive CoCl_2_, or cryptotanshinone treatment were used as the negative control. All mice femurs were isolated and ground in the presence of liquid nitrogen. The tissue debris was soaked in guanidinium thiocyanate (GTC) buffer (Omega Bio-tek). Total RNA was extracted with EZNA Total RNA Kit (Omega). Total RNA (1 μg) was reverse-transcribed to cDNA using the First Strand cDNA Synthesis Kit (Takara). Quantitative real-time PCR was performed using All-In-One qPCR Mix (GeneCopoeia) and a Bio-Rad CFX 96 Real-time PCR cylcer. Glyceraldehyde 3-phosphate dehydrogenase (GAPDH) was used as a housekeeping gene. All the primers used for quantitative real-time PCT are listed in Table 1.

**Table 1:** Primer sequences

Gene	Acc. No	Primer sequence (5′ → 3′)	Size (bp)
*Hif-1α*	NM_176958	F: GTCCCAGCTACGAAGTTACAGC	136
		R: CAGTGCAGGATACACAAGGTTT	
*Alp*	NM_001081082	F: GGCCATCTAGGACCGGAGA	79
		R: TGTCCACGTTGTATGTCTTGG	
*Opn*	NM_001204203	F: ATCTCACCATTCGGATGAGTCT	79
		R: TGTAGGGACGATTGGAGTGAAA	
*Runx2*	NM_001146038	F: GACTGTGGTTACCGTCATGGC	84
		R: ACTTGGTTTTTCATAACAGCGGA	
*Col1α1*	NM_007742	F: GCTCCTCTTAGGGGCCACT	91
		R: ATTGGGGACCCTTAGGCCAT	
*Osx*	NM_146065	F: ATCATTGCAGATCAAACGCCT	135
		R: AGCACCCTTTTTCTCATCGTC	
*Ocn*	NM_031368	F: CACTCCTCGCCCTATTGGC	112
		R: CCCTCCTGCTTGGACACAAAG’	
*Opn*	NM_026493	F: GAAGAGCAAAAAGCGAAACTGG	153
		R: TTGGCTGCTTGGTGGAATGT	
*Vegf*	NM_005429	F: GAGGAGCAGTTACGGTCTGTG	96
		R: TCCTTTCCTTAGCTGACACTTGT	
*Gapdh*	NM_008085	F: AATGGATTTGGACGCATTGGT	213
		R: TTTGCACTGGTACGTGTTGAT	

### Western blot assay

For western blot analysis, MSCs (2 × 10^5^ cells/well) were seeded in 6-well plates and cultured in osteogenic media. Cells were treated with CoCl_2_ (50 μM) for 1, 3, 5 and 7 days. Cryptotanshinone (10 μM) or DMSO was added to the cells in the appropriate groups for the duration of the culture. Cell lysates were extracted on day 7 [18, 22]. Total protein was estimated using the BCA Protein Assay (Thermo Scientific). Total protein (20 μg) was separated by 10% SDS-PAGE (Biotech) and transferred to polyvinylidene difluoride membrane (Roche). The membranes were blocked with 5% skimmed milk (Biosharp) absorbed in 10% tris-buffered saline with 0.1% tween 20 (TBST; Gibco) at room temperature for 1 h. Then, the membranes were incubated on a shaker for 8 h at 4 °C with one of the primary antibodies: anti-HIF-1α (Santa), anti-ALP (Abcam), anti-Osx (Abcam), anti-Runx2 (Abcam), anti-Col1α1 (Santa) and anti-GAPDH (Protech). The membranes were then incubated with secondary antibody (Abbkine) and absorbed in TBST for 1 h at room temperature. Blots were visualized, and the relative density of each blot was determined using Image J software 1.49 (NID).

### ALP staining and ALP activity

For ALP staining and activity, MSCs (1 × 10^5^ cells/well) were seeded in 24-well plates and cultured in osteogenic medium. Cells were treated with CoCl_2_ (50 μM) for 1, 3, 5 and 7 days. Cryptotanshinone (10 μM) or DMSO was added to the cells in the appropriate groups for the duration of the culture. The ALP staining and activity measurements were conducted on day 7 [18, 22].

### Alizarin red assay

To analyze the mineralized matrix, MSCs (1 × 10^5^ cells/well) were seeded in 24-well plates and cultured in osteogenic medium. Cells were treated with CoCl_2_ (50 μM) for 1, 3, 5 and 7 days. Cryptotanshinone (10 μM) or DMSO was added to the cells in the appropriate groups for the duration of the culture. All the cultures were continued till day 14 in presence of osteogenic medium. The matrix-mineralized nodules were stained with alizarin red on day 14. Cultures were washed 3 times with PBS, fixed with 95% ethanol for 15 min, and washed 3 times with distilled water. The cells were stained with 0.1% alizarin red S (Sigma) dissolved in 0.1 M Tris-HCl buffer for 30 min. A light microscope was used to visualize the alizarin red-stained mineralized matrix. Quantification involved dissolving the mineralized matrix with 1% cetylpyridinium chloride and measuring the absorbance of the dissolved matrix at 562 nm using a microplate reader.

### In vivo bone defect healing

The femoral defects were prepared based on findings from our previous research [24]. The anesthetic agent was a mixture of 1.5 mg/ml xylazine and 10 mg/ml ketamine. It was intraperitoneally injected at a dose of 0.1 ml/10 g body weight. A longitudinal lateral thigh incision was made to expose the femur. A perforated defect was drilled in the middle of the femur using a 0.8 mm straight shank twist drill (GB/T6135.2, Shanghai Tool Works) at 3000 rpm attached to a grinder set (P-500-6A, Slite). Then a 1.4-mm straight shank twist drill (Shanghai Tool Works) was used to extend the defect. To avoid thermal injury, saline irrigation was used. The surgical area was flushed with saline solution to remove the bone chips. The incision was closed in layers with sterile silk suture. For postoperative analgesia, 50 μl buprenorphine hydrochloride at a concentration of 0.04 mg/ml was injected every 12 h subcutaneously for 2 days.

Mice in the blank control group received no treatment. The treatment consisted of daily intraperitoneal injection with: CoCl_2_ (13.5 mg/kg body weight) for the CoCl_2_ and CoCl_2_ + inhibitor groups; cryptotanshinone (5 mg/kg body weight) for the CoCl_2_ + inhibitor and inhibitor groups; or 0.1% DMSO absorbed in PBS (control group).

In each group, 5 mice were randomly assigned to each time point (1, 3 or 5 weeks post operation). For RNA isolation and qPCR analysis, samples from 15 mice (3 mice/group, total 5 groups) were used as described in the section on gene expression analysis. Mice were killed 1, 3 or 5 weeks after surgery. Femora were fully dissected and fixed in 4% PFA for 48 h.

### Micro-CT analysis

The femora were fixed in 4% paraformaldehyde and scanned using a μ-CT 50 imaging system (Scanco Medical). The tube was set at 70 kV, 85 mA with 20 μM resolution. Newly formed bone volume and trabecular parameters were quantified in a 2-mm^3^ cube that completely contained the bone defect area.

### Histology and immunohistochemistry study

Fixed bone tissues (*n* = 5/group) were embedded in paraffin and sliced into 5 μM thick tissue sections using a microtome (Thermo Fisher Scientific). The tissue sections were deparaffinized and stained with hematoxylin and eosin (H&E). Newly formed bone and osteoids were visualized under a light microscope. For immunohistochemistry, tissue sections were stained according to the standard protocol [22]. The primary antibodies were anti-HIF-1α (CST, 1:400 dilution), anti-pSTAT3 (CST, 1:200 dilution), anti-ALP (Abcam, 1:400 dilution). The universal immunoperoxidase (HRP) ABC kit (ZSGB bio) was used to visualize the secondary antibody.

### Statistical analysis

All the in vitro and in vivo experiments were performed 5 times (*n* = 5). Data analysis was performed using a one-way analysis of variance (ANOVA) followed by Bonferroni’s multiple comparison test using GraphPad Prism 7.0 software. In all cases, the selected significance level was *p* < 0.05.

## Results

### Cellular hypoxia suppressed MSC proliferation but enhanced osteogenic differentiation

**Fig. 1:**
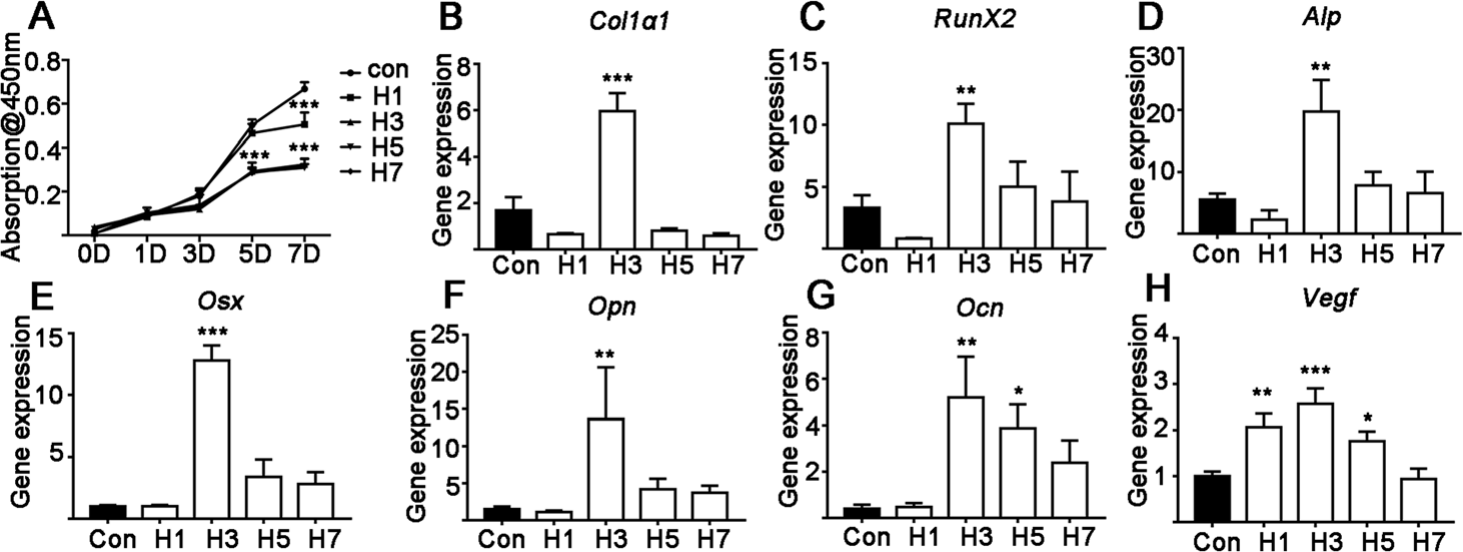
The effect of continuous hypoxia for 1, 3, 5 and 7 days on MSC proliferation and gene expression of osteogenic markers. **a** MSC proliferation on days 1, 3, 5 and 7 of culture including 1, 3, 5 or 7 days of hypoxia. **b**-**h** Osteogenic and *Vegf* gene expression on day 7. Data are the means ± SD from 5 independent experiments, *n* = 5. Significant effect of the treatment: *p < 0.05, **p < 0.01 and ***p < 0.001. H1, H3, H5 and H7 represent hypoxia for 1, 3, 5 and 7, respectively, whule 0D, 1D, 3D, 5D and 7D represent 0, 1, 3, 5 or 7 total days of culture, respectively. *Col1α1*: collagen I alpha1, *Runx2:* Runt-related transcription factor 2, *Alp*: alkaline phosphatase, *Osx:* osterix, *Ocn*: osteocalcin and *Vegf:* vascular endothelial growth factor

The hypoxia simulated by CoCl_2_ treatment significantly suppressed MSC proliferation. One day of hypoxia (i.e., 1 day of CoCl_2_ treatment followed by 6 days of incubation without CoCl_2_) did not show an impact on MSC proliferation after 1, 3 or 5 days of culture, but 1.34-fold inhibition was observed on day 7 (Fig. 1a). Interestingly, hypoxia for 3, 5 and 7 days showed similar inhibitory effect on cell proliferation after 5 and 7 days of culture (Fig. 1a).

Osteogenic gene expression, ALP staining and ALP activity in MSCs were analyzed on day 7. Hypoxia for 3 days respectively upregulated *Col1α1*, *Runx2*, *Alp*, *Osx, Opn*, *Ocn* and *Vegf* gene expression by 3.12-, 3.35-, 4.12-, 14.29-, 8.35-, 12.1- and 2.61-fold compared to the control group (Fig. 1b–j). Hypoxia for 5 days enhanced only *Ocn* and *Vegf* gene expression (respectively by 9.07- and 1.75- fold compared to the control group). Hypoxia for 1 day enhanced *Vegf* gene expression by 2.05-fold compared to the control group, but did not affect the expressions of other osteogenic markers (Fig. 1h). Interestingly, continuous simulation of hypoxia for 7 days did not affect all the expressions of all the osteogenic markers tested (Fig. 1b–j).

**Fig. 2:**
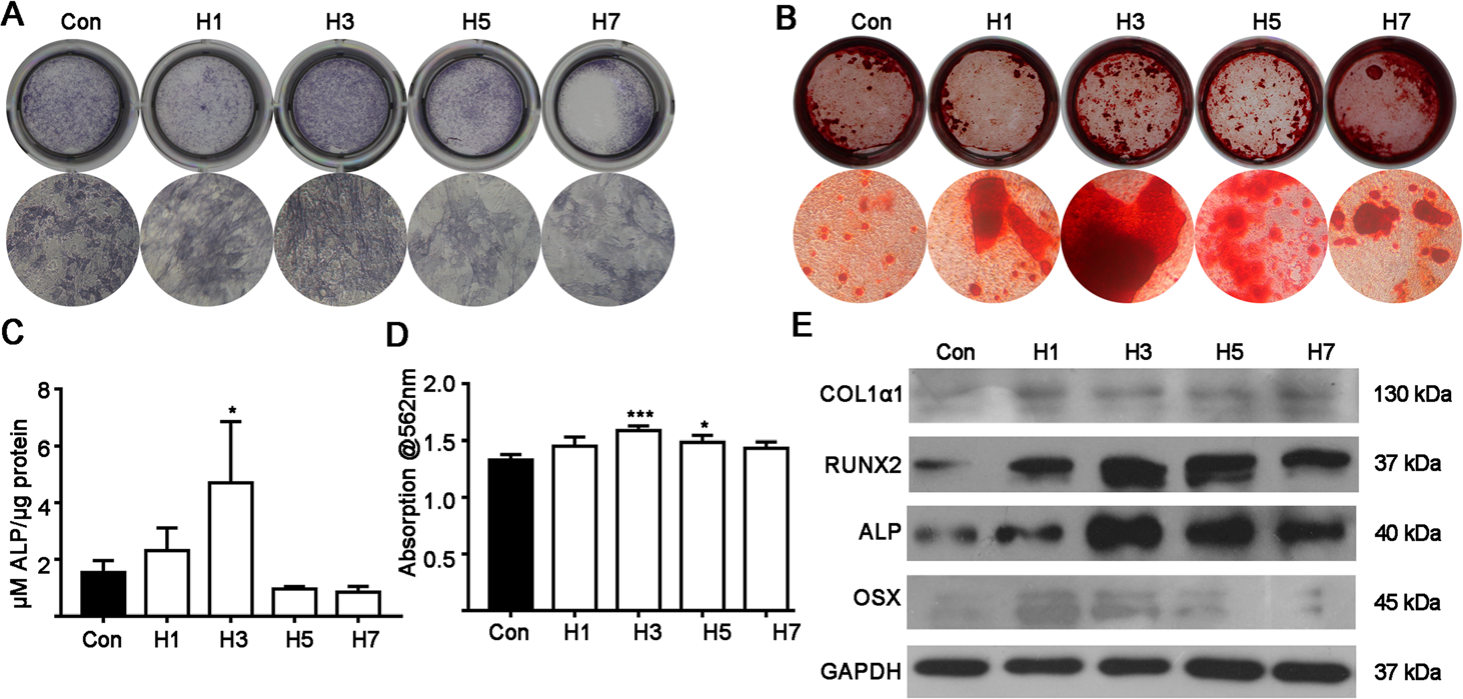
The effect of continuous hypoxia for 1, 3, 5 and 7 days on ALP activity and matrix mineralization. **a** ALP staining on day 7. **b** Matrix mineralization (alizarin red staining) on day 14. **c** ALP activity on day 7. **d** Quantitative analysis of alizarin red staining. **e** Osteogenic differentiation marker (protein) expression. Data from quantitative analysis are the means ± SD from 5 independent experiments, *n* = 5. Significant effect of the treatment: *p < 0.05, **p < 0.01 and ***p < 0.001

Hypoxia for 3 days yielded the strongest ALP and alizarin red staining (Fig. 2a and c). Similarly, hypoxia for 3 days enhanced ALP activity by 2.92- fold compared to the control group (Fig. 2c). Quantification of the mineralized matrix showed that hypoxia for 3 and 5 days respectively promoted matrix mineralization by 1.18-, and 1.09-fold compared to the control group (Fig. 2d).

COL1α1, OSX, RUNX2, and ALP protein expressions were analyzed using western blot assay on day 7. Hypoxia for 1, 3, 5 and 7 days respectively enhanced COL1α1 protein expression by 1.50-, 1.55-, 1.41- and 1.47- fold (Fig. 2e and Additional file 1: Figure S1A). Hypoxia for 1, 3, 5 and 7 days respectively enhanced RUNX2 protein by 2.67-, 4.05-, 2.97- and 2.29- fold (Fig. 2e and Additional file 1: Figure S1A). Hypoxia for 1, 3, 5 and 7 days respectively enhanced ALP protein expression by 1.81-, 4.97-, 2.68- and 1.91- fold (Fig. 2e and Additional file 1: Figure S1A). Similarly, hypoxia for 1, 3, 5 and 7 days respectively enhanced OSX protein expression by 1.84-, 2.71-, 2.25- and 2.08- fold (Fig. 2e and Additional file 1: Figure S1A). Hypoxia for 3 days showed the highest effect on osteogenic marker protein expression, which concurs with the results for mRNA expression.

### STAT3 inhibitor reversed hypoxia-induced STAT3 phosphorylation and osteogenic differentiation

**Fig. 3:**
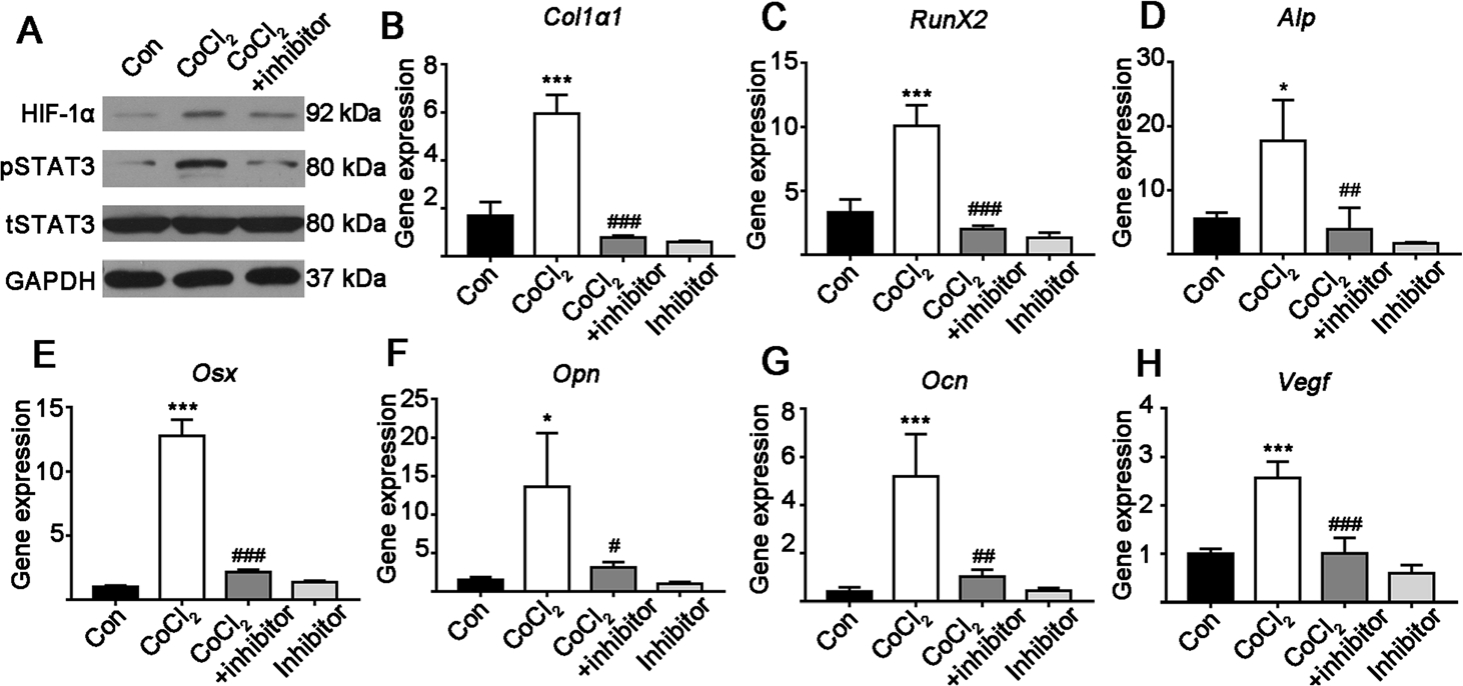
The effect of continuous hypoxia for 3 days with or without a STAT3 inhibitor. **a** Representative western blot images. **b**-**h** The effect of 3 days of hypoxia with or without a STAT3 inhibitor on osteogenic marker and *Vegf* gene expression in MSCs. Data of quantitative analysis are the means ± SD from 5 independent experiments, *n* = 5. Significant effect of the treatment compared to the control group: *p < 0.05 and ***p < 0.001; and the CoCl_2_ group: ^#^*p* < 0.05, ^##^*p* < 0.01 and ^###^*p* < 0.001

Cellular hypoxia enhanced HIF-1α expression by 1.81-fold. The STAT3 inhibitor did not alter hypoxia-induced HIF-1α expression (Fig. 3a and Additional file 1: Figure S2A). Hypoxia did not affect the total STAT3 expression (Fig. 3a). Hypoxia robustly enhanced (5.46-fold) STAT3 phosphorylation and the STAT3 inhibitor reduced this effect by 3.10-fold (Fig. 3a and Additional file 1: Figure S2B).

**Fig. 4:**
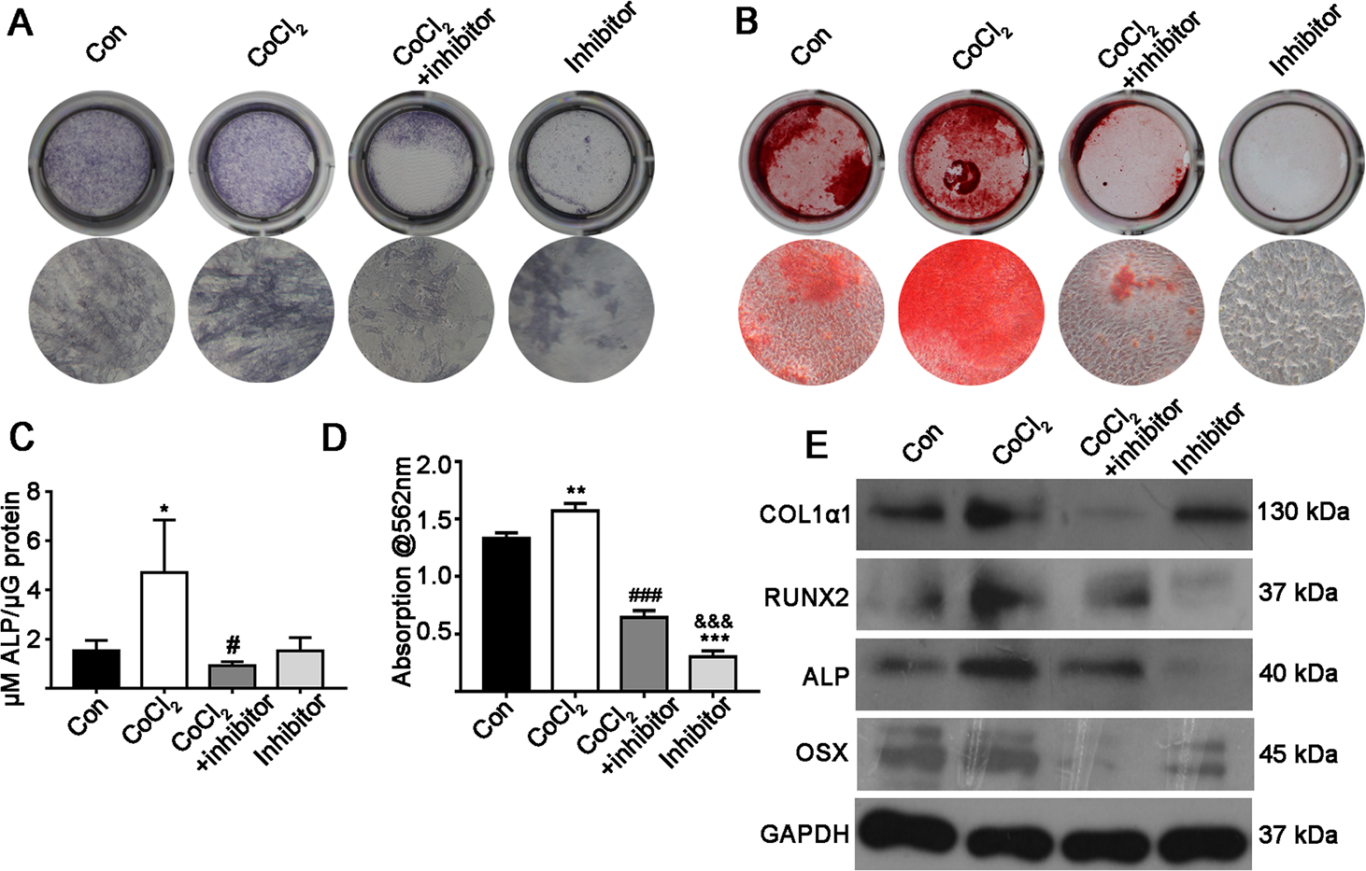
The effect of continuous hypoxia for 3 days on days 1, 3, 5 and 7 of culture with or without a STAT3 inhibitor. **a** ALP staining on day 7. **b** Matrix mineralization (alizarin red staining) on day 14. **c** ALP activity on day 7. **d** Quantitative analysis of alizarin red staining. **e** Osteogenic differentiation marker (protein) expression. Data of quantitative analysis are the means ± SD from 5 independent experiments, *n* = 5. Significant effect of the treatment compared to the control group: *p < 0.05, **p < 0.01 and ***p < 0.001; the CoCl_2_ group: ^#^*p* < 0.01, ^###^*p* < 0.001; and the CoCl_2_ + inhibitor group: ^&&&^*p* < 0.001. Inhibitor: STAT3 inhibitor

Since hypoxia for 3 days showed the highest effect on the osteogenic differentiation of MSCs, we choose this culture condition to analyze the effect of STAT3 inhibitor on hypoxia-induced osteogenic differentiation. STAT3 inhibitor respectively suppressed hypoxia-induced *Col1α1*, *Runx2*, *Alp*, *Osx, Opn*, *Ocn* and *Vegf* gene expression by 6.13-, 4.87-, 5.67-, 6.56-, 4.31-, 5.41- and 2.63-fold (Fig. 3b–h). STAT3 inhibitor alone did not affect the expression of osteogenic genes compared to the control group (Fig. 3b–h). STAT3 inhibitor reduced hypoxia-induced ALP protein expression and ALP activity (5.38-fold; Fig. 4a and c). STAT3 inhibitor strongly reduced (2.37-fold) hypoxia-induced matrix mineralization (Fig. 4b and d). Similarly, STAT3 inhibitor reduced matrix mineralization by 2.08- and 4.51-fold respectively compared to the results for the CoCl_2_ + inhibitor and control groups.

Western blot data analysis showed that STAT3 inhibitor respectively reduced hypoxia-induced COL1α1, RUNX2, ALP and OSX protein expressions by 4.56-, 1.67-, 1.34- and 1.78-fold (Fig. 3e and Additional file 1: Figure S3A–D). STAT3 inhibitor reduced OSX protein expression by 1.89-fold compared to the control group (Additional file 1: Figure S3D). However, STAT3 inhibitor did not affect the expressions of the other osteogenic proteins tested compared to the control group. RUNX2, ALP and OSX protein expression in STAT3 inhibitor group were respectively suppressed by 3.23-, 3.02- and 2.35-fold compared to the hypoxia + STAT3 inhibitor group (Additional file 1: Figure S3B–D).

### Hypoxia enhanced bone regeneration and STAT3 inhibitor impaired this effect

**Fig. 5:**
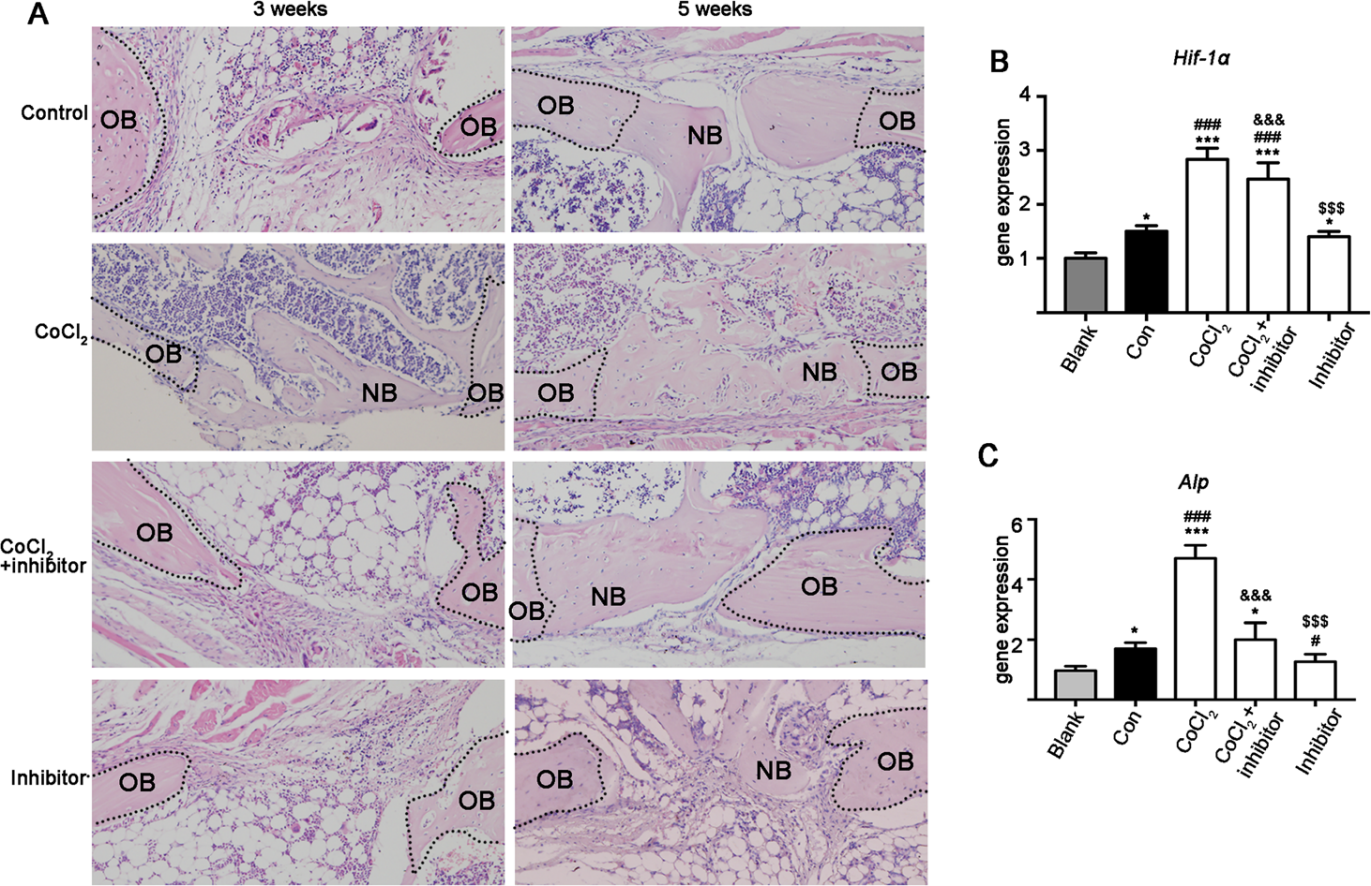
Histology of bone defects from mouse femurs and associated *Hif-1α* and *Alp* gene expressions. **a** Representative images of mouse femoral bone defect histological section (H & E staining). **b** and **c** – *Hif-1α* and *Alp* gene expression in mouse femoral bone defects on day 7. Data of quantitative analysis are the means ± SD, *n* = 5. Significant effect of the treatment compared to the blank group: *p < 0.05 and ***p < 0.001; the control group: ^#^*p* < 0.05, ^###^*p* < 0.001; the CoCl_2_ group: ^&&&^*p* < 0.001; and the CoCl_2_ + inhibitor group: ^$$$^*p* < 0.001. Inhibitor: STAT3 inhibitor, OB: original bone, NB: newly formed bone, Red arrow: defect area with failure to regenerate bone

Histological images showed more newly formed bone in the bone defect area at week 3 in the CoCl_2_ group compared to the images for the control, hypoxia + STAT3 inhibitor, and STAT3 inhibitor groups (Fig. 5a). Similarly, the bone defect area was filled with newly formed bone in the CoCl_2_ group at week 5. However, a clear bone defect gap was observed in the control, hypoxia + STAT3 inhibitor, and STAT3 inhibitor groups (Fig. 5a).

### Hypoxia upregulated *Hif-1α* and *Alp* mRNA expression in bone defect femora and STAT3 inhibition reversed this effect

To investigate the possible interaction between hypoxia and STAT3 signaling during osteogenesis and bone defect healing, we analyzed *Hif-1α* and *Alp* mRNA expression in mice femoral bone defects treated with CoCl_2_ and/or STAT3 inhibitor. *Hif-1α* and *Alp* mRNA expression were upregulated in the femurs of all the bone defect groups compared to the results for the blank control group (Fig. 5b and c). CoCl_2_-induced hypoxia further upregulated *Hif-1α* and *Alp* expression by 1.81- and 2.77-fold, respectively (Fig. 5b and c). STAT3 inhibitor reduced hypoxia-induced *Hif-1α* and *Alp* expression by 1.15- and 2.30-fold, respectively (Fig. 5b and c). The STAT3 inhibitor did not affect *Hif-1α* expression but suppressed the *Alp* expression by 1.31-fold compared to the control group (Fig. 5c).

### CoCl_2_-simulated hypoxia promoted bone defect healing and STAT3 inhibitor reversed this effect

**Fig. 6:**
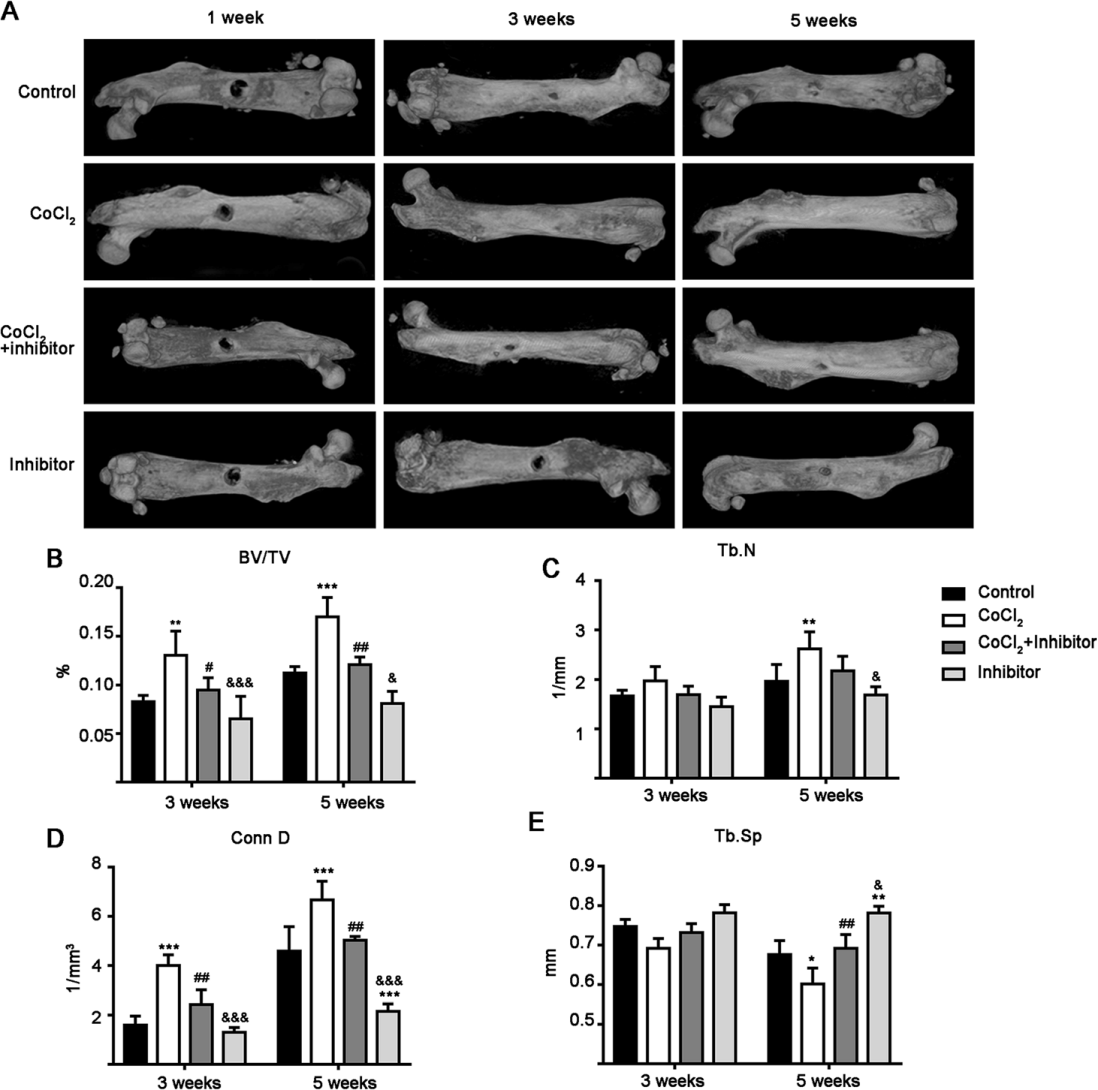
Images and trabecular parameters for bone defects. **a** Representative μ-CT images of mouse femurs with bone defects. **b**-**e** Quantitative analysis of bone trabecular parameters in the bone defect area. Data of quantitative analysis are the means ± SD from 5 independent experiments, *n* = 5. Significant effect of the treatment compared to the control group: *p < 0.05, **p < 0.01 and ***p < 0.001; the CoCl_2_ group: ^#^*p* < 0.05 and ^##^*p* < 0.01; and the CoCl_2_ + inhibitor group: ^&^*p* < 0.05 and ^&&&^*p* < 0.001. Inhibitor: STAT3 inhibitor

μ-CT and X-ray images showed that CoCl_2_ promoted femoral bone defect healing at week 3 and 5 compared to the control group (Fig. 6a and Additional file 1: Figure S4). Interestingly, the STAT3 inhibitor reversed hypoxia-induced bone defect healing at week 3 and 5 (Fig. 6a and Additional file 1: Figure S4). Moreover, STAT3 inhibitor reduced bone defect healing compared to the control, CoCl_2_ and CoCl_2_ + STAT3 inhibitor groups (Fig. 6a and Additional file 1: Figure S4).

Similar effects of CoCl_2_ and STAT3 inhibitor were shown by the newly formed bone trabecular parameters at week 3 and week 5 (Fig. 6b–e). CoCl_2_ treatment enhanced BV/TV and Conn D levels by 1.51- and 2.44-fold, respectively, at week 3 compared to the control. STAT3 inhibitor reduced the CoCl_2_-induced impact on BV/TV and Conn D levels by 1.37- and 1.64-fold, respectively (Fig. 6b and d). Similarly, STAT3 inhibitor reduced BV/TV and Conn D levels by 1.28- and 1.27-fold, respectively, compared to the control group at week 3 (Fig. 6a and d). CoCl_2_ treatment enhanced BV/TV, Tb. N and Conn D levels by 1.49-, 1.45- and 1.46-fold, respectively, at week 5 compared to the control group (Fig. 6b–e). STAT3 inhibitor reduced the CoCl_2_-induced impact on BV/TV and Conn D levels by 1.38- and 1.31- fold, respectively, at week 5 (Fig. 6b–e). Moreover, STAT3 inhibitor reduced Conn D levels by 2.08-fold compared to the control (Fig. 6b–e), and reduced BV/TV, Tb. N, and Conn D levels by 1.49-, 1.25- and 2.27-fold, respectively, compared to those for the CoCl_2_ + inhibitor group at week 5 (Fig. 6b–e). Hypoxia suppressed Tb. Sp levels by 1.38-fold at week 5 compared to the control group (Fig. 6e). STAT3 inhibitor reversed hypoxia-mediated suppression at week 5 (Fig. 6e). Moreover, the STAT3 inhibitor group enhanced Tb. Sp levels by 1.36- and 1.30-fold at week 5 compared to the control and CoCl_2_ + inhibitor groups, respectively (Fig. 6e).

### Hypoxia upregulated HIF-1α, pSTAT3 and ALP protein expression in the bone defect area and STAT3 inhibitor reversed this effect

**Fig. 7:**
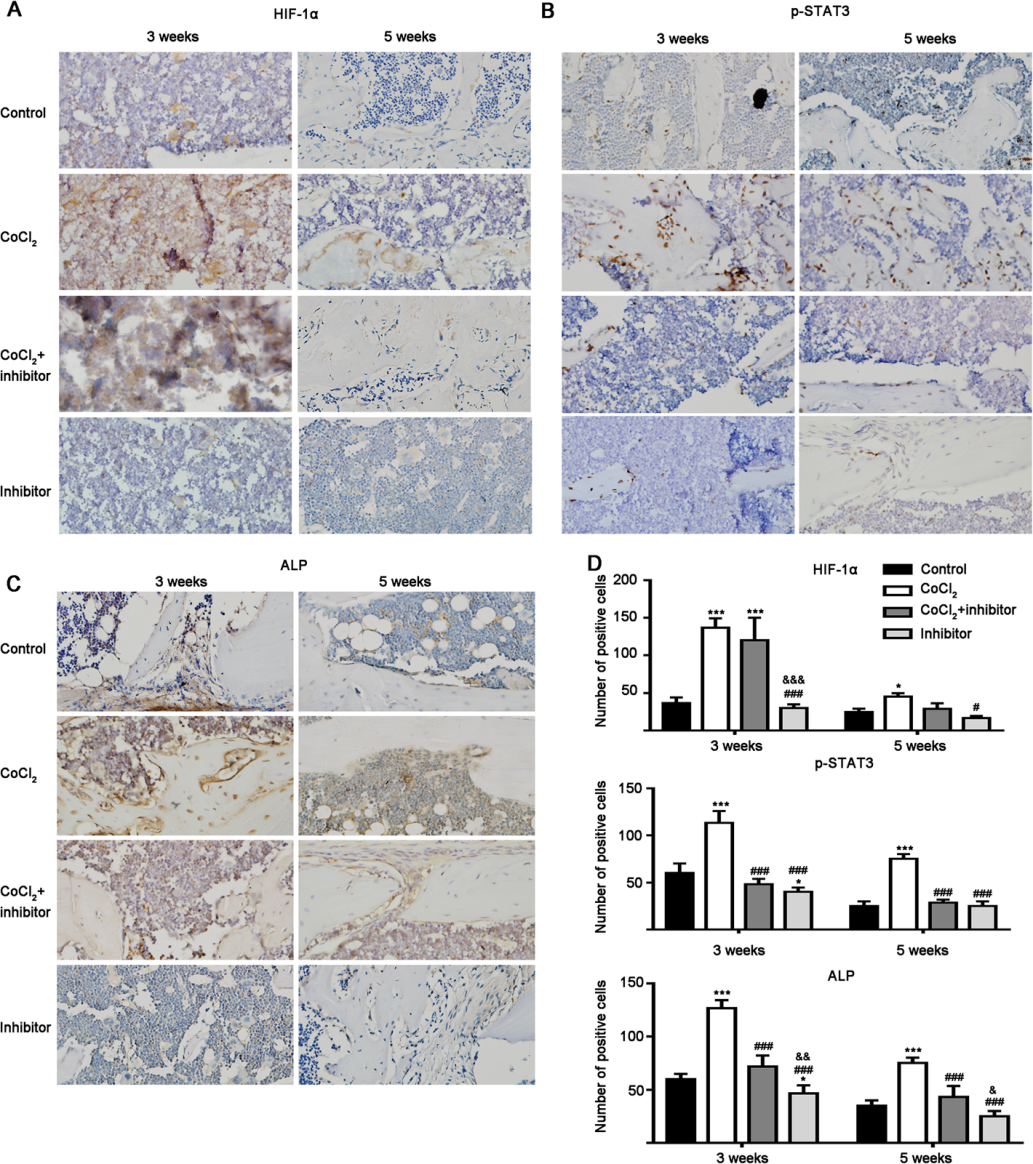
Representative immunohistochemistry images for proteins in bone defect tissue sections. **a** – HIF-1α. **b** – p-STAT3. **c** – ALP. **d** – Quantitative analysis of HIF-1α, p-STAT3 and ALP protein expression in bone defect area. Significant effect of the treatment compared to the control group: *p < 0.05 and ***p < 0.001; the CoCl_2_ group: ^#^*p* < 0.05 and ^###^*p* < 0.001; and the CoCl_2_ + inhibitor group: ^&^*p* < 0.05 and ^&&^*p* < 0.01. Inhibitor: STAT3 inhibitor

Immunohistochemistry images showed stronger immunostaining (brown and light brown color) of HIF-1α, p-STAT3 and ALP at week 3 compared to the staining at week 5 in all the groups tested (Fig. 7a–c). The CoCl_2_-treated group showed stronger immunostaining of HIF-1α, p-STAT3 and ALP compared to all other groups at week 3 and 5.

Quantitative analysis of the immunohistochemistry of tissue section showed 3.32- and 1.74-fold higher expression of HIF-1α in the hypoxic group compared to the control group at week 3 and 5, respectively (Fig. 7a and d). STAT3 inhibitor did not affect CoCl_2_-induced HIF-1α expression at week 3 and 5. The CoCl_2_ group showed 1.87- and 2.85-fold higher expression of pSTAT3 compared to the control group at week 3 and 5, respectively (Fig. 7a and d). STAT3 inhibitor reversed the hypoxia-induced pSTAT3 upregulation at week 3 and 5. Similarly, the CoCl_2_-group showed 2.02- and 1.97-fold higher expression of ALP compared to the control group at week 3 and 5, respectively (Fig. 7a and d). STAT3 inhibitor reduced the hypoxia-induced ALP expression by 1.73- and 1.70-fold at week 3 and 5, respectively.

## Discussion

The crosstalk (interaction) between cellular hypoxia and STAT3 signaling during bone defect healing has not been reported on yet. In this study, we found that short-term (3-day) cellular hypoxia enhanced the osteogenic differentiation of MSCs and bone defect healing, and that inhibition of STAT3 signaling reversed this effect. Moreover, cellular hypoxia upregulated *Vegf*, HIF-1α and pSTAT3 expression during in vitro osteogenic differentiation and bone defect healing. The STAT3 inhibitor neutralized this effect. These findings suggest that the interaction between hypoxia and STAT3 signaling is significant in bone defect healing.

We analyzed the effects of the different durations of hypoxia on MSC proliferation during a 7-day culture. Hypoxia for 3, 5 and 7 days resulted in a similar level of inhibition on MSCs proliferation on day 7. The effects of different duration of hypoxia on osteogenic marker expression was also evaluated on day 7. Three days of hypoxia robustly enhanced the expressions of most osteogenic markers at the mRNA and protein levels on day 7, while 1, 5 and 7 days of hypoxia did not have this effect (Fig. 1). This result was further confirmed by the highest ALP activity (on day 7) and matrix mineralization (on day 14) being found for MSCs exposed to hypoxic conditions for 3 days (Fig. 2).

It is well known that hypoxia in early-stage bone defect healing triggers the healing process. However, it has also been reported that continuous hypoxia inhibits osteogenic differentiation of precursor cells [25, 26] via activation of Notch1 signaling [26] and inhibition of Runx2 [27]. Osathanon et al. had reported that continuous treatment with CoCl_2_ (50 μM) for 7 days does not affect OCN gene expression, ALP activity or matrix mineralization in human periodontal ligament stem cell culture [25]. Xu et al. reported the inhibitory effect of continuous hypoxia for 7 days or more on the osteogenic differentiation of MSCs [26]. Moreover, Genetos et al. reported that 48 h hypoxia activates Wnt signaling and suppresses sclerostin expression in osteoblasts [28]. Activated Wnt signaling induces osteogenesis and high sclerostin expression inhibits it.

During the early stage of bone defect healing, hypoxia enhances the migration of osteogenic and angiogenic precursor cells as well as osteogenesis and angiogenesis. Newly formed vessels around the bone defect eliminate the hypoxic condition and the healing process continues. During embryo development, hypoxia is essential for vascularization of the placenta and embryo. Similarly, endochondral ossification during fetal bone development requires a hypoxic environment. Hypoxia-mediated endochondral ossification also plays a role in large size bone defect healing [29]. Tissue engineering techniques utilizing hypoxia are being developed to repair large bone and cartilage defects. Hypoxic conditions (3% oxygen) in bioreactors enhance chondrogenesis and cartilage matrix component formation [30]. Moreover, intermittent hypoxia has been reported to promote hippocampal neurogenesis and provide antidepressant-like effects in adult rats [31].

The bone defect healing process also indicates the importance of the period of hypoxia. In this study, continuous hypoxia inhibited most osteogenic differentiation markers, including Runx2. Our results showed that hypoxia for too short (1 day) or too long (5 and 7 days) a period failed but for 3 days robustly enhanced the osteogenic differentiation of precursor cells. This suggests hypoxia has a crucial optimal duration that induces bone regeneration in the healing process.

The activation of STAT3 signaling has been reported to enhance the osteogenic differentiation of precursor cells and bone defect healing [19, 32]. Gao et al. had reported that hypoxia enhances STAT3 signaling in synovial fibroblasts [33]. However, a few studies have investigated the interaction between hypoxia and STAT3 signaling during the osteogenic differentiation of precursor cells. In this study, CoCl_2_ treatment enhanced HIF-1α protein expression in MSCs (Fig. 3a), which indicates that the CoCl_2_ treatment in MSC culture could induce cellular hypoxia. Since cellular hypoxia enhanced STAT3 phosphorylation and a STAT3 inhibitor reduced this effect (Fig. 3a), we further investigated the effect of the STAT3 inhibitor on hypoxia-induced osteogenic differentiation and bone defect healing. Interestingly, inhibition of STAT3 dramatically reversed the stimulatory effect of hypoxia on osteogenic differentiation of MSCs (Figs. 3 and 4). These findings indicate that hypoxia-mediated activation of STAT3 promotes osteogenic differentiation of MSCs. This is the first study to report on STAT3-mediated osteogenic differentiation of MSCs and matrix mineralization under hypoxic conditions.

Osteogenesis–angiogenesis coupling plays a vital role in bone regeneration during bone defect healing [11, 34]. VEGF is a known pro-angiogenic as well as a pro-osteogenic factor with well-established function on endothelial cells and osteoblasts during bone defect healing [2, 11, 35]. Osteoblast lineage cell-derived VEGF has been reported as a key player in the stages of the bone repair process, i.e., osteogenic differentiation, angiogenesis and osteogenesis–angiogenesis coupling [11]. Hypoxia mimicking biomaterials reportedly promote bone defect healing via the upregulation of VEGF signaling [8, 36]. Wang et al. reported that STAT3 signaling mediates VEGF production in MSCs [37]. In this study, we found that cellular hypoxia enhanced VEGF expression and the STAT3 inhibitor reversed this effect (Figs. 1h and 3h). These results indicate the possible role of STAT3 signaling in the VEGF-mediated angiogenesis and the bone defect healing process. However, further in vitro and in vivo study focusing on the interaction between STAT3 and VEGF signaling during hypoxia-induced bone defect healing is necessary to prove this hypothesis.

Disrupted blood circulation creates a hypoxic environment in the bone defect area. Hypoxia increases HIF-1α protein expression in precursor cells in the bone defect [38]. We found that HIF-1α expression was upregulated in the injured femur, and STAT3 inhibitor reversed this effect (Figs. 5 and 7). Interestingly, expression of the early osteogenesis marker ALP was upregulated in the injured femur. The CoCl_2_ treatment further upregulated ALP expression and inhibition of STAT3 nullified this effect (Figs. 5 and 7). Moreover, STAT3 phosphorylation was upregulated in precursor cells in the defect area at weeks 3 and 5, and STAT3 inhibitor reversed this effect (Fig. 7). CoCl_2_ treatment enhanced new bone formation and bone defect healing, and inhibition of STAT3 reduced this effect.

Most of our results from in vitro studies were supported by the results from the in vivo study on bone defect healing. Activation of HIF-1α in osteoblast lineage cells had been reported to enhance bone regeneration [6]. Similarly, Durand et al. reported that hypobaric hypoxia accelerates bone defect healing in mice [7]. STAT3 activation in mesenchymal stem cells had been reported to enhance osteogenic differentiation and in vivo bone formation [39–41]. Moreover, STAT3 activation in peripheral blood mononuclear cells had been reported to promote bone fracture healing [19]. Our findings show that hypoxia promotes osteogenesis and bone defect healing via activation of STAT3 signaling in precursor cells.

In this study, we investigated the effect of different durations of cellular hypoxia on osteogenic marker expression at the mRNA and protein levels. More prominent osteogenic marker ALP staining, ALP activity, and matrix mineralization were also investigated. The possible role of STAT3 signaling in hypoxia-mediated osteogenesis was investigated both in vitro and in vivo. In terms of future verification, there are a few possible approaches. We used CoCl_2_ to simulate hypoxia in vitro, and these results could be verified in the future with cell cultures incubated in a hypoxic environment. The results from mice MSCs should be verified with human MSCs or MSCs from STAT3 knockout mice. Similarly, a future study using MSC-specific STAT3 knockout mice for bone defect healing is recommended.

## Conclusions

Both hypoxia and STAT3 signaling are involved in the osteogenic differentiation of precursor cells and bone defect healing. However, the role of the interaction between hypoxia and STAT3 signaling in bone defect healing is not clear. In this study, we found that cellular hypoxia inhibited MSC proliferation but enhanced osteogenic differentiation. Hypoxia for 3 days showed the highest anabolic effect on the osteogenic differentiation of MSCs. Hypoxia upregulates STAT3 phosphorylation and VEGF expression in MSCs. The STAT3 inhibitor reversed this effect. Hypoxia facilitated bone regeneration and bone defect healing in mouse femur bone defects. Inhibition of STAT3 signaling reduced the hypoxia-induced osteogenic differentiation of MSCs in vitro, and bone regeneration and healing in mice femoral defects, suggesting a possible role of STAT3 signaling in hypoxia-mediated osteogenic differentiation of precursor cells and bone defect healing.

## Supplementary information

**Additional file 1: Figure S1.** Quantitative analysis of western blots for protein expression in BMSCs on day 7 (Fig. 2e). A – Col1α1. B – RUNX2. C – ALP. D – OSX. H1, H3, H5 and H7 represent hypoxia for 1, 3, 5 and 7 days, respectively. Inhibitor: STAT3 inhibitor. Data of quantitative analysis are the means ± SD from 5 independent experiments, *n* = 5. Significant effect of treatment, *p < 0.05, **p < 0.01 ***p < 0.001 compared with the control group. **Figure S2.** Quantitative analysis of western blots for protein expression in BMSCs after 3 h of culture (Fig. 3a). A – HIF-1α/GAPDH. B – pSTAT3/tSTAT3. Data of quantitative analysis are the means ± SD from 3 independent experiments, *n* = 5. Inhibitor: STAT3 inhibitor. Significant effect of treatment, *p < 0.05, **p < 0.01 ***p < 0.001 compared with the control group; ^###^*p* < 0.001 compared with the CoCl_2_ group. **Figure S3.** Quantitative analysis of western blots for protein expression in BMSCs on day 7 (Fig. 4e). A – Col1α1. B – RunX2. C – ALP. D – Osx. H1, H3, H5 and H7 represent hypoxia for 1, 3, 5 and 7 days, respectively. Inhibitor: STAT3 inhibitor. Data of quantitative analysis are the means ± SD from 5 independent experiments, *n* = 5. Significant effect of treatment compared to the control group: *p < 0.05, **p < 0.01 and ***p < 0.001; the CoCl_2_ group: ^###^*p* < 0.001; and the CoCl_2_ + inhibitor group: ^&&&^*p* < 0.001. **Figure S4.** Representative X-Ray images of mice femurs with bone defects. Inhibitor: STAT3 inhibitor.

## Data Availability

All the data are included within this manuscript. The raw data of the study are available from the corresponding author on reasonable request.

## Abbreviations

BMSCs: Bone marrow stromal cells
CoCl_2_: Cobalt chloride
HIF-1α: Hypoxia-inducible-factor 1-alpha
JAK2: Janus kinase 2
MSCs: Mesenchymal stem cells
STAT3: Signal transducer and activator of transcription-3
VEGF: Vascular endothelial growth factor
