# Supplementary material for: Cellular hypoxia promotes osteogenic differentiation of mesenchymal stem cells and bone defect healing via STAT3 signaling
Source: Cell Mol Biol Lett. 2019 Dec 3;24:64. doi: 10.1186/s11658-019-0191-8 (PMC6889321; doi:10.1186/s11658-019-0191-8)
Supplement: Supplementary file 1 — Additional file 1: Figure S1. Quantitative analysis of western blots for protein expression in BMSCs on day 7 (Fig. 2e). A – Col1α1. B – RUNX2. C – ALP. D – OSX. H1, H3, H5 and H7 represent hypoxia for 1, 3, 5 and 7 days, respectively. Inhibitor: STAT3 inhibitor. Data of quantitative analysis are the means ± SD from 5 independent experiments, n = 5. Significant effect of treatment, *p < 0.05, **p < 0.01 ***p < 0.001 compared with the control group. Figure S2. Quantitative analysis of western blots for protein expression in BMSCs after 3 h of culture (Fig. 3a). A – HIF-1α/GAPDH. B – pSTAT3/tSTAT3. Data of quantitative analysis are the means ± SD from 3 independent experiments, n = 5. Inhibitor: STAT3 inhibitor. Significant effect of treatment, *p < 0.05, **p < 0.01 ***p < 0.001 compared with the control group; ###p < 0.001 compared with the CoCl2 group. Figure S3. Quantitative analysis of western blots for protein expression in BMSCs on day 7 (Fig. 4e). A – Col1α1. B – RunX2. C – ALP. D – Osx. H1, H3, H5 and H7 represent hypoxia for 1, 3, 5 and 7 days, respectively. Inhibitor: STAT3 inhibitor. Data of quantitative analysis are the means ± SD from 5 independent experiments, n = 5. Significant effect of treatment compared to the control group: *p < 0.05, **p < 0.01 and ***p < 0.001; the CoCl2 group: ###p < 0.001; and the CoCl2 + inhibitor group: &&&p < 0.001. Figure S4. Representative X-Ray images of mice femurs with bone defects. Inhibitor: STAT3 inhibitor. [file 11658_2019_191_MOESM1_ESM.docx]

**Additional file**

**Title:** Cellular hypoxia promotes osteogenic differentiation of mesenchymal stem cells and bone defect healing via STAT3 signaling

**Authors:** Xin Yu, Qilong Wan, Xiaoling Ye, Yuet Chen, Janak L. Pathak, Zubing Li

**Journal:** Cellular and Molecular Biology Letters

**Figure S1**

**
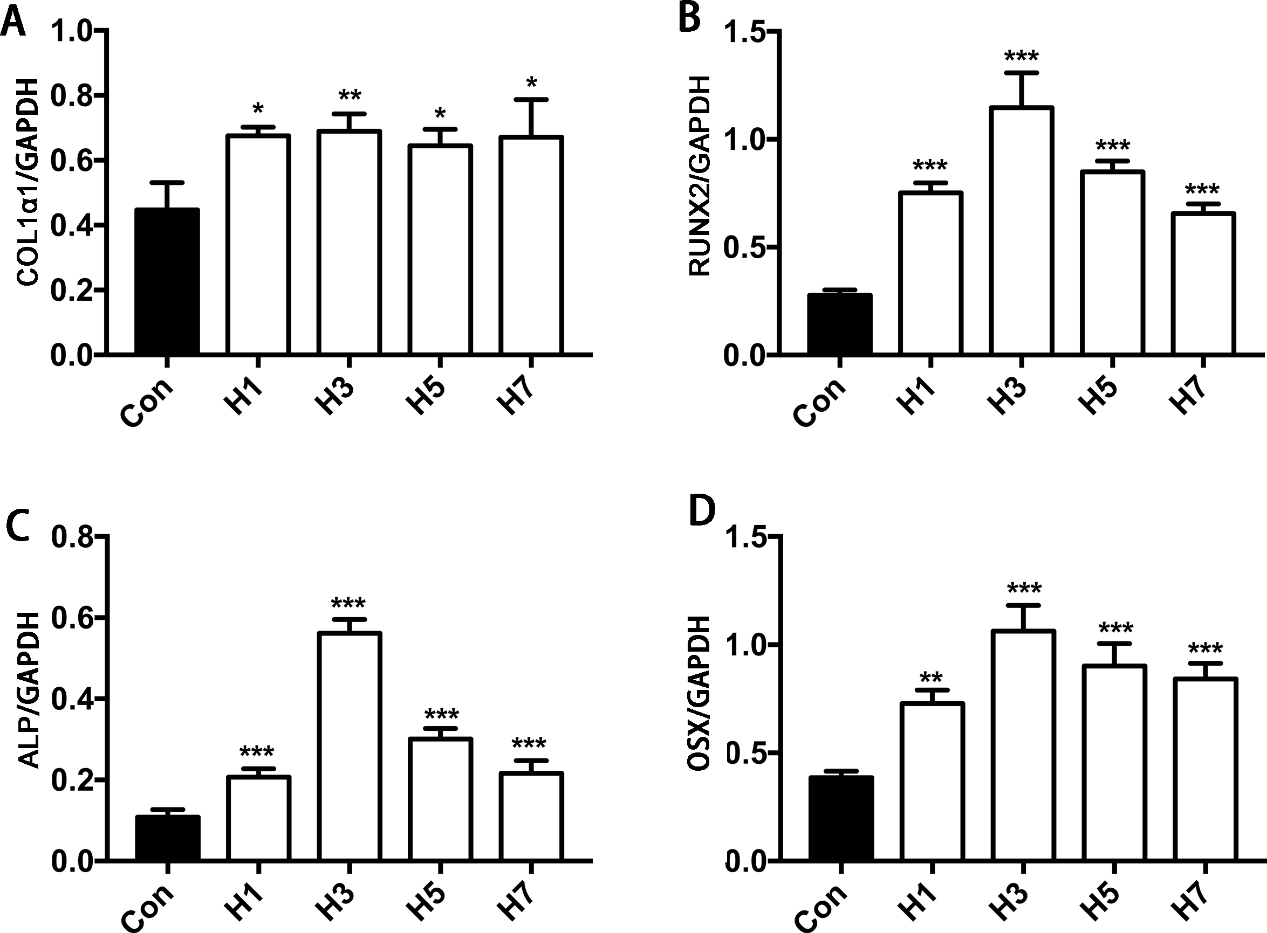
**

**Figure S1.** Quantitative analysis of western blots for protein expression in BMSCs on day 7 (Figure 2E). A – Col1α1. B – RUNX2. C – ALP. D – OSX. H1, H3, H5 and H7 represent hypoxia for 1, 3, 5 and 7 days, respectively. Inhibitor: STAT3 inhibitor. Data of quantitative analysis are the means ± SD from 5 independent experiments, n = 5. Significant effect of treatment, *p < 0.05, **p < 0.01 ***p < 0.001 compared with the control group.

**Figure S2**

**
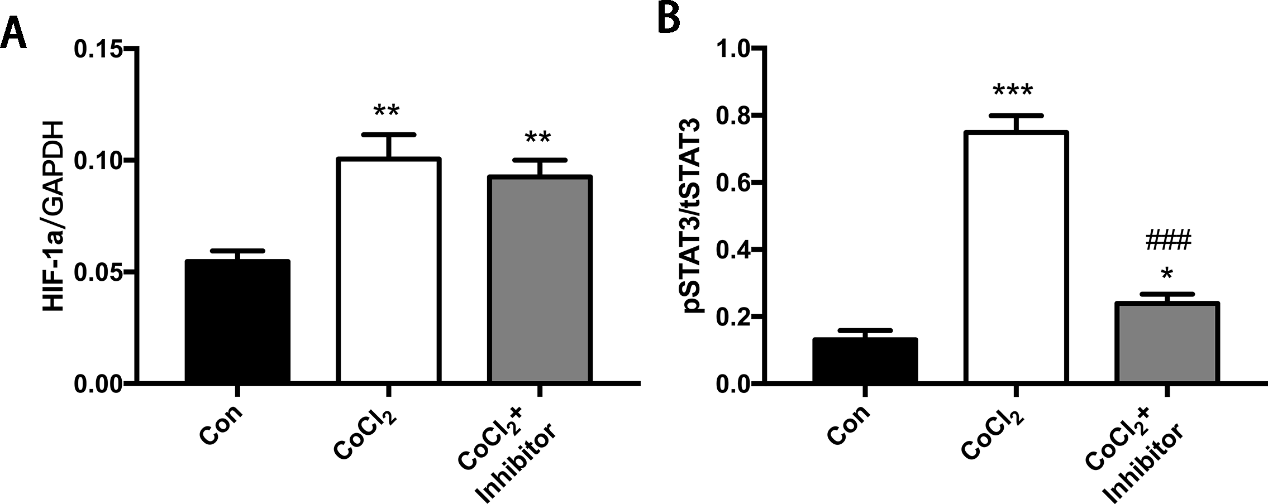
**

**Figure S2.** Quantitative analysis of western blots for protein expression in BMSCs after 3 h of culture (Figure 3A). A – HIF-1α/GAPDH. B – pSTAT3/tSTAT3. Data of quantitative analysis are the means ± SD from 3 independent experiments, n = 5. Inhibitor: STAT3 inhibitor. Significant effect of treatment, *p < 0.05, **p < 0.01 ***p < 0.001 compared with the control group; ^###^p < 0.001 compared with the CoCl_2_ group.

**Figure S3**

**
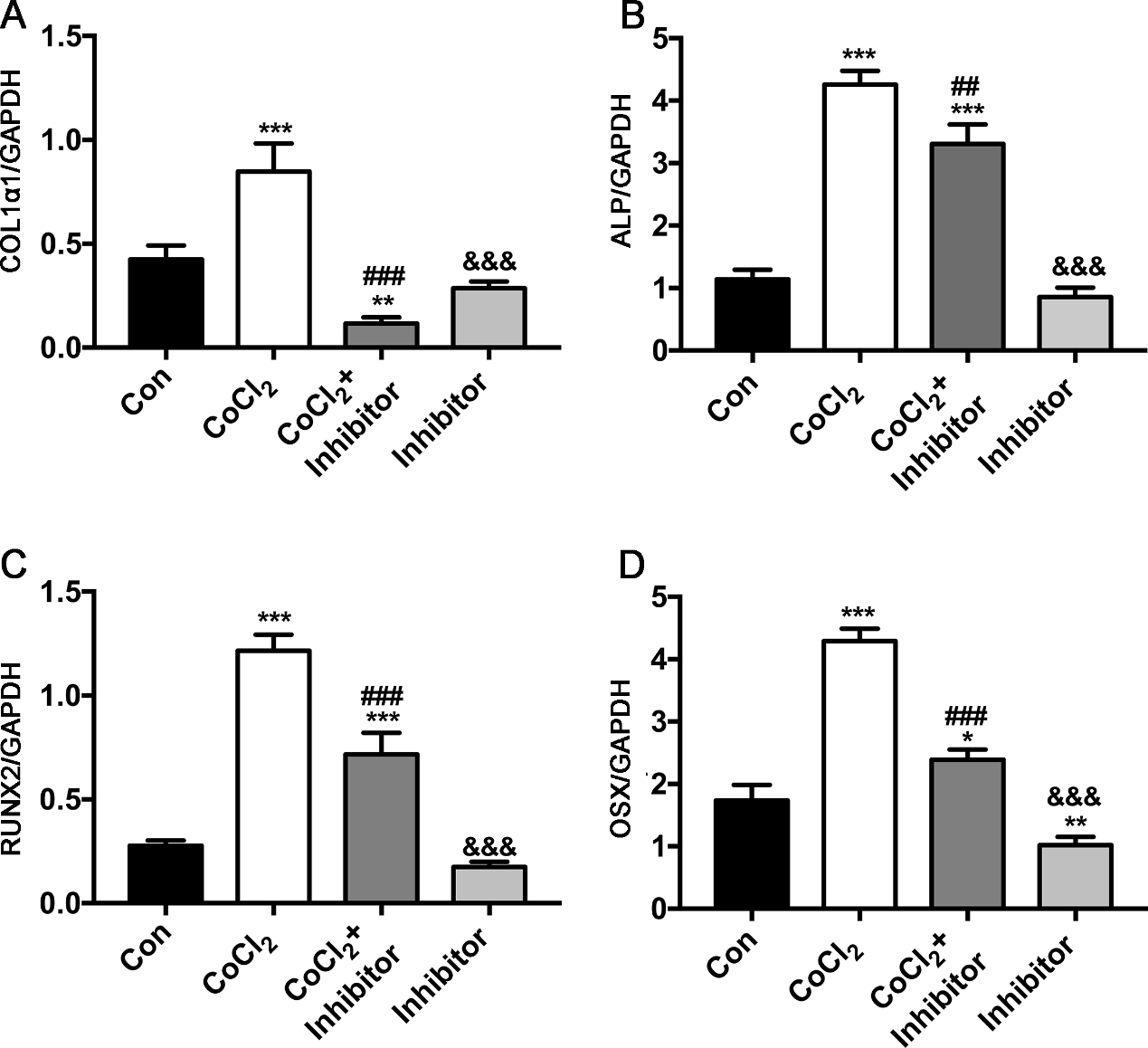
**

**Figure S3.** Quantitative analysis of western blots for protein expression in BMSCs on day 7 (Figure 4E). A – Col1α1. B – RunX2. C – ALP. D – Osx. H1, H3, H5 and H7 represent hypoxia for 1, 3, 5 and 7 days, respectively. Inhibitor: STAT3 inhibitor. Data of quantitative analysis are the means ± SD from 5 independent experiments, n = 5. Significant effect of treatment compared to the control group: *p < 0.05, **p < 0.01 and ***p < 0.001; the CoCl_2_ group: ^###^p < 0.001; and the CoCl_2_ + inhibitor group: ^&&&^p < 0.001.

**Figure S4**


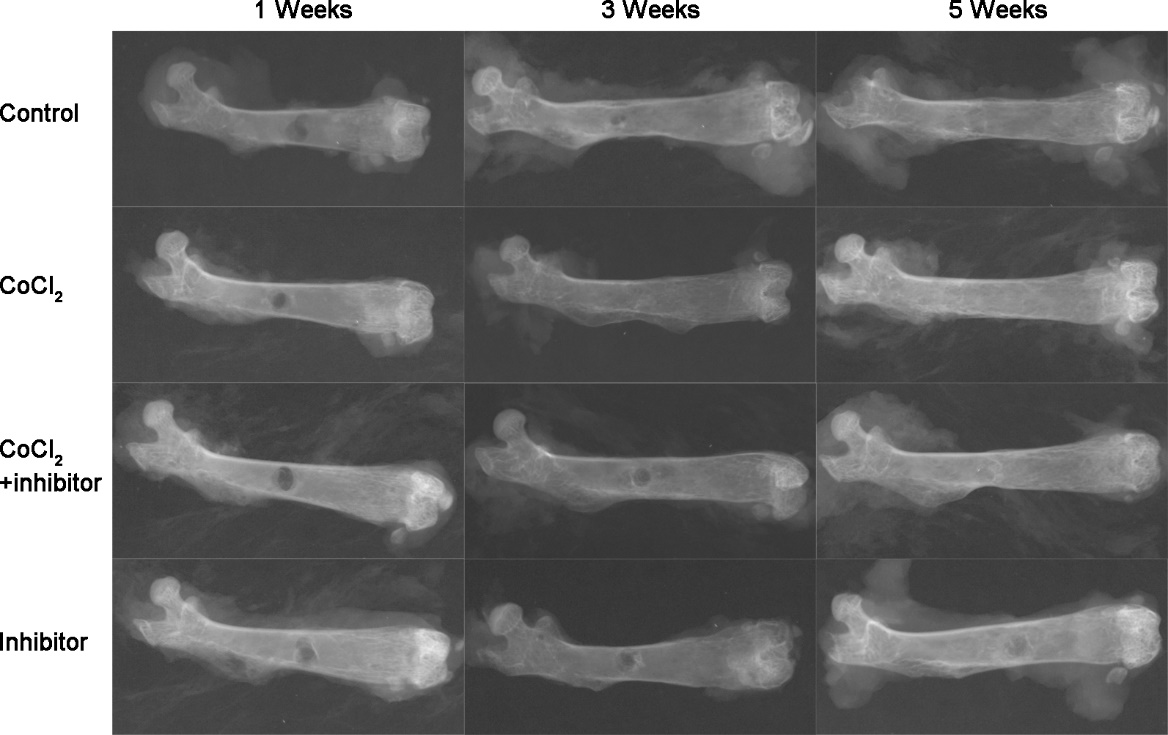


**Figure S4. Representative X-Ray images of mice femurs with bone defects.** Inhibitor: STAT3 inhibitor.
